# The complete mitogenome of *Acropora nana* (Cnidarian: Acroporidae)

**DOI:** 10.1080/23802359.2017.1365638

**Published:** 2017-08-17

**Authors:** Xinming Liu, Xinqing Zheng, Chung-Der Hsiao, Rongcheng Lin, Xiaofeng Shi, Wentao Niu

**Affiliations:** a Guangxi Academy of Oceanography, Nanning, China;; b Third Institute of Oceanography, State Oceanic Administration, Xiamen, China;; c Department of Bioscience Technology, Chung Yuan Christian University, Chung-Li, Taiwan;; d Center for Biomedical Technology, Chung Yuan Christian University, Chung-Li, Taiwan;; e Center for Nanotechnology, Chung Yuan Christian University, Chung-Li, Taiwan

**Keywords:** *Acropora nana*, mitogenome, next generation sequencing

## Abstract

*Acropora nana* belongs to *Cnidaria* phylum, *Anthozoa* class, and staghorn coral groups. Like other corals, *Acropora* could be an indicator for environmental changes and with great conservational values. In this study, the complete mitogenome sequence of *A. nana* (Cnidarian: Acroporidae), has been decoded for the first time by low coverage whole genome sequencing method. The overall base composition of *A. nana* mitogenome is 25.0% for A, 13.8% for C, 24.4% for G, and 36.9% for T, and has low GC content of 38.2%. The assembled mitogenome, consisting of 18,480 bp, has unique 13 protein-coding genes (PCGs), three transfer RNAs, and two ribosomal RNAs genes. The *A. nana* has one big intron insert in ND5 gene. The complete mitogenome of *A. nana* provides essential and important DNA molecular data for further phylogenetic and evolutionary analysis for stony corals.

Coral reefs are known as home for many marine species. Climate change causes rising ocean temperature, ocean acidification, and more frequent storm. Rising ocean temperature can lead to coral bleaching. Like other corals, *Acropora* could be an indicator for environmental changes. *Acropora nana* can be found in Taiwan, China, Australia, Indonesia, and Japan sea water. Like other corals, *A. nana* feeds on tiny zooplankton, however, it receives most of the nutrients from single-celled algae ‘zooxanthellae’ which live within its tissues. Stable environment for zooxanthellae to live is provided by *A. nana*, in returns it receives nutrients from photosynthesis by zooxanthellae. This mutualism symbiosis restricts *A. nana* to live in shallow, clear, and warm water, where photosynthesis is possible, and makes it to grow quickly and form large reef structure. The colonies are quite delicate and readily breaking apart if disturbed, but the colonies live in high wave current sea (Veron and Stafford-Smith 2000). *A. nana* is morphologically similar to *A. valida* in wave washed habitats and it can be distinguished by its thicker branchlets and chunkier radial corallites. This issue makes the classification and molecular methods are needed in this coral identification.

Samples of *A. nana* (TIOSOA-LCC-20150704-Acro.Na) were collected from Houhai coral reefs in Sanya (18°16′32.58″N 109°44′10.74″E), Hainan Province. We used next generation sequencing to perform low-coverage whole genome sequencing according to our previous protocol (Shen et al. 2016). Initially, the raw next generation sequencing reads were generated from HiSeqX Ten (Illumina, San Diego, CA). About 0.13% raw reads (32,090 out of 25,616,912) were *de novo* assembly by using commercial software (Geneious V9, Auckland, New Zealand) to produce a single, circular form of complete mitogenome with about an average 261× coverage.

The complete mitogenome of *A. nana* was 18,480 bp in size (GenBank KY094488) and its overall base composition is 25.0% for A, 13.8% for C, 24.4% for G, and 36.9% for T, and have GC content of 38.2%, showing 99% identities to *A. divaricate* (GenBank KF448537) after BLAST search against NCBI nr/nt database. The protein coding, rRNA and tRNA genes of *A. nana* mitogenome were predicted by using DOGMA (Wyman et al. 2004), ARWEN (Laslett and Canback 2008), MITOS (Bernt et al. 2013) tools and manually inspected. The complete mitogenome of *A. nana* includes unique 13 protein-coding genes (PCGs), three transfer RNA (trnA, trnM, and trnW) genes and two ribosomal RNA (rrnL and rrnS) genes. All PCGs, tRNA, and rRNA genes were encoded on R-strand. It is interesting to note that one big intron (12,116 bp) is inserted in ND5 gene. It is important to note that six PCGs started with ATG codon (ATP6, ATP8, CYTB, ND2, ND3, and ND4L), one with ATT codon (COX1), one with GTG codon (COX2), three with TTG codon (COX3, ND4, and ND5), two with ATA codon (ND1 and ND6), and one with TTA codon (ND5). Nine of 13 PCGs are inferred to terminate with TAA (ATP6, ATP8, COX1, COX2, ND1, ND2, ND4, ND4L, ND5, and ND6) and four PCGs with TAG (CYTB, COX3, ND3, and ND5). Among 13 PCGs, the longest open reading frame length is ND5 gene (1704 bp), whereas the shortest is ATP8 gene (219 bp).

To validate the phylogenetic position of *A. nana*, we used MEGA6 software (Tamura et al. 2013) to construct a maximum likelihood tree with 100 bootstrap replicates and Kimura 2-parameter (K2P) model containing complete mitogenomes of 14 species derived from *Acropora* genus of Scleractinia. *Montipora cactus* derived from *Montipora* genus was used as outgroup for tree rooting. Result shows *A. nana* is closed to *A. divaricata* (Figure 1) with a very high identity of 99% (18,468/18,481) and a very small K2P distance of 0.0007. The average K2P distance within *Acropora* genus is 0.0027, while the average K2P distance between *Acropora* sp. with *Montipora cactus* is 0.4723. This result clearly demonstrates the mitogenome sequences between *Acropora* sp. are very closely each other. In conclusion, the complete mitogenome of the *A. nana* deduced in this study provides essential and important DNA molecular data for further phylogenetic and evolutionary analysis for stony coral.

**Figure 1.**
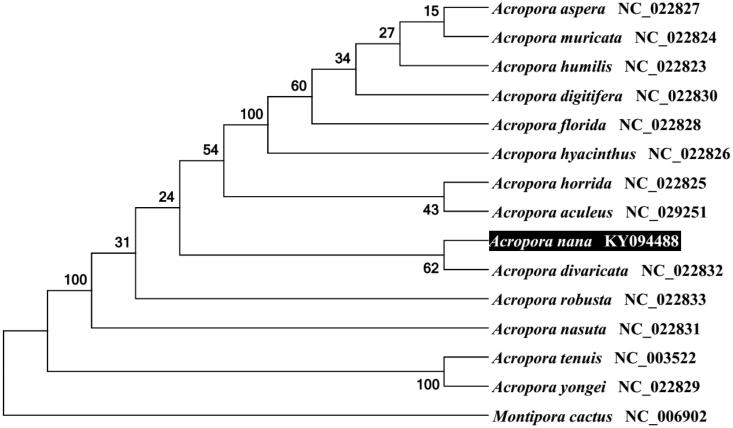
Molecular phylogeny of *A. nana* and related species based on complete mitogenome. The complete mitogenomes is downloaded from GenBank and the phylogenetic tree is constructed by maximum likelihood method with 100 bootstrap replicates. The gene's accession number for tree construction is listed behind the species name.
